# Dr. Slack’s Address

**Published:** 1848-04

**Authors:** Elijah Slack


### Dr. Slack's Address.

INTRODUCTORY LECTURE *delivered in the Ohio College of Dental Surgery, November*, 1847.

BY ELIJAH SLACK, M. D.

GENTLEMEN:

The time for the resumption of the duties of the several departments in Dental instruction, is arrived, and as is customary, you expect of us some introductory remarks to the Chemical course. We may not offer much inviting, but of that you present are to judge. As far as practicable, we shall endeavor in our discussions to introduce *principles* based on authenticated facts. After these few passing words, we trust, you will hear us patiently, while we introduce your old, respectable, and never to be abandoned friend, *Chemistry*, whose fame, in Virgil's language, "is known above the stars," and whose character is extending from year to year.

It may be defined, that science, which examines and arranges compounds, decomposes them, and presents the laws of decomposition and recomposition. From this passing definition, the extensive range of our subject may be discovered. It is occupied only with matter, of course, with every material object connected with the earth, whether solid, liquid, or airy form; with light, heat and electricity, however foolish and visionary many theories of the existence and operations of the latter may be. With all their *imponderability*, they constitute the most powerful and efficient materials in the catalogue of chemical substances.*

It is hardly necessary to say, that the progress of our subject is rapid. From a few crude and badly arranged facts and dogmas, the latter not even called principles, in less than a century, Chemistry, has become a noble science, whose title is conceded and to whose influence, the learned and ignorant alike pay homage.

The success in art and science, in material things, as the gospel in morals, has introduced to the civilized world a new era. Who, that examines the course of things, for the last fifty years, does not perceive the improvement in almost every department of business? To what science is this progress owing more than to the subject we discuss? It is this which has made us familiar with the elements of matter and its compounds, that has not only drawn immensely on inorganic nature, so as to reduce her almost to order, but with scrutinizing eye, has looked into the deep mysteries of organic nature, so as to develope the laws of nutrition, increase, maturity and decay, in both vegetables and animals. This latter department, by the efforts of giant minds, laboring incessantly in a field hitherto almost untrodden, within a few years, has furnished mines of the richest treasure, as the reward of the practical agri-

* We say "chemical substances," notwithstanding the vast amount of learned mystery about a "subtile elastic ether" existing in the universe, making up the absolute "plenum" of former schools. After the efforts of Young, Airy, Somerville, Brewster, Herschel, Melloni and others, the existence of *this ether* has never been proved; not even by the retarded motions of cometary bodies. It exists as an elaborated *figment* of the imagination. Light, heat and electricity, result agreeably to these notions from the *vibrations* of this "elastic ether." In other words, motion, a mere property of matter, constitutes light and color in all varieties, even the colored figure of external objects on the retina, caloric in all its modifications and electricity, wherever found. Having followed *this fancy*, through many of its windings, in different volumes, I can see nothing like substantial science in it. Believe who choose. If the internal chemical structure of bodies, as far as molecules and crystalization are concerned, were once well understood, the Newtonian position, of the materiality of light, caloric and electricity, would triumphantly explain all phenomena, even the recondite ones of polarized light.

culturist or studious physician, whose aim is always to benefit his fellow creatures.

To return to the notice of a *new era* above, who does not see *this* fully established in the wonderful march of improvement, in our own and in other countries? Is it not to Chemistry we stand more especially indebted? Has she not furnished to the skillful mechanic, almost volcanic power, in the application of steam to his well adjusted machinery? Has not this power given impulse to manufactories in their thousand varied forms, producing results the richest, finest and most desirable, so as to add to every well organized government, wealth in an untold amount? In 1785, cotton was first grown in flower pots, as a curiosity, on the sea-coast of Georgia; at this time, the United States produces yearly, enough of this fabric, to furnish a full suit to every man, woman and child on the Globe—though the population be one thousand millions—and the machinery of the world, propelled mostly by steam, converts, annually, this vast staple into yarns and cloth, for the benefit of untold millions. The man of sixty, remembers well, how difficult a voyage across the Atlantic was considered, now, by steam power, space is almost annihilated. Our steam ships are but simple ferry-boats, and the once formidable Atlantic, one of our largest ferries. Turn the eye to our improved railroad highways, connecting immense countries, and we see a small specimen of mechanics and Chemistry. We say small, for others are employed, much exceeding in grandeur, perhaps not in power. We behold the steam car and train, with giant power, outstripping the fleetest animal of the forest, and literally leaping over space, so as to bring the most distant points of a nation, in immediate vicinity. Fifty years ago the appellation of *madman* would have been given to him, who had dared to predict such results. To Chemistry, also, is to be voted a triumph (though she will no doubt achieve greater results) for the Electrical Telegraph, now operating in our city. News by the lightning line, is common, yet strange language. The velocity of the electrical fluid, if it could, like light, be accurately measured, would probably equal two hundred thousand miles in a second of time. The wonderful phenomenon in these days of wonder, has been presented, of two individuals, seven hundred miles apart, maintaining a conversation as if seated in the same room—and the same will be realized for greater distances, even round the Globe, whenever telegraph lines thus extensive, be constructed.

These amazing results of Chemistry* have given impulse to every department of art and science, so that the world is literally moving onward, to veritable greatness, with a rapidity never anticipated by our immediate forefathers.

If every profession be progressive, and every art be proceeding to the goal of comparative perfection, what shall be said of the Dentist and regular Physician, whose professions well secured, is the most difficult to be obtained, in the catalogue of professions. Shall he, because his subjects and investigations are abstruse, fold his arms in listlessness? Shall he cease to make efforts? or shall he pass over to some new *ism*, *eclectic* or *opathy*, at present so fashionable? In order, the more effectually to deceive the public and be popular, shall he denounce calomel and a few standard medicines of the regular type, made injurious by the quack prac-

* We have classed steam and electricity among chemical substances, and it is believed for good reasons. 1st. Because they are chemical results, and not mechanical. They, however, from peculiar properties, exert mechanical force, and thus have been arranged under mechanical philosophy. 2nd. In regard to steam—it is constituted of common water and caloric, exhibiting great elastic force as a gas. Oxygen and caloric make the chemical gas, called oxygen; sulphur and hydrogen, with caloric, form sulphuretted hydrogen; carbon, oxygen and caloric, carbonic acid gas; but the latter are said to be permanently elastic, while steam is condensible. The expression *permanently elastic*, is merely relative; for atmospheric air has been condensed into a liquid, and carbonic acid gas, not only into a liquid, but a solid. The great value of steam consists in its elastic force and easy condensibility, and power of boiling and heating at a distance from the source of heat, and who does not consider boiling and heating chemical results? 3d. Electricity, if produced by an amalgamed rubber on a common glass, electric has been arranged under mechanics. "Why?" If electricity belong to that science, surely caloric ought to belong to the same!! We know not the modus operandi of the amalgamed rubber, or simply of the hand or a silk rubber, or of continued friction in boring metals, wood, or the passage of steam out of orifices in an insulated boiler, yet they all produce electricity, and the latter seven or eight times as much as the others. (See Harper's Somerville, page 280.) Galvanic electricity, most certainly results from chemical action, and is a powerful chemical agent, but the various kinds are near relatives of the one great principle. Shall we not call it a chemical substance, producing chemical as well as mechanical action?

tice of the administrator? Fye! for shame. Let him stand up to his work like a man. Let him lay deep and broad the foundations of a regular medical education, and he can laugh at the innovations of empirics, who hope by a cry of poison, and by a few carricature handbills, to entrench themselves in ill-gotton places, and thus deceive the public, who for their credulity deserve imposition, till they learn to beware of such hollow-hearted demonstrations. It is plain, that the old and regular course of practice, where it is thorough, is the only effectual method of reaching disease, and furnishing aid to the great family of the afflicted.

It is here to be conceded, and we design honestly to contend for this point, that much reform is required in the regular schools of medicine in order to lay in the dust the herd of *isms* and *opathies*, that so extensively trifle with the public favor. It is to the want of a thorough education, in the most responsible profession, except the gospel ministry, to which we are to refer the many innovations on the regular practice.

In the first place, Medical schools, rivals to each other, are too numerous. A great temptation is furnished to faculties, to graduate, yearly, as many pupils as possible, in order to secure by the numbers sent forth, an influence, and to swell the list of diplomas, to compare advantageously with sister institutions. In this way many have been honored in different schools, with a degree, who, in all fairness, ought not to have been graduated till a subsequent period, and some of them not at all.

Second. Our Legislatures, supposed to be the guardians of every thing good, have, in our opinion, very unwisely multiplied charters for Medical schools, (additions to which are made almost every session) till the whole business is overdone, and superficial qualifications pretty certainly secured to the profession and detriment to the public for a generation to come. We advocate on this subject, nothing of aristocratic principles, but simply thorough preparation.

Third. The candidates for a medical profession, in the regular or *ism* or *opathy* schools, are too numerous for the public demand; so that each aspirant may secure a patronage sufficient for support.

Hence a multitude of new systems are introduced, which cannot secure permanence, unless "Ishmael like" war is declared against the regular practice, and the most sweeping denunciations uttered, not only against the former but even against one another. By this course the public is disturbed, and they who have nothing to lose, oftentimes come in for a share of attention, which, under other circumstances, would not have been awarded. These persons act warily on the principles of the artizan and merchant; that opposition is the life of trade, and in this manner, innovate upon their neighbors. The procedure might be justified, provided the employer could judge as well of the abilities of a *new coined* or *ism* practitioner, as of a manufactured article, or the price of goods, provisions, or stocks; but here any analogy ends, and the public are seriously injured.

Fourth. In our regular schools, the preponderating influence of professors and students, has been directed to certain branches of a medical education, to the almost entire neglect of others, superlatively fundamental. In this manner, every student sent out with such preparations and views, must necessarily, at least to the extent intimated, have too frequently the very seal of unpreparedness, and be a solemn drawback on the regular practice, and those schools, if we can find such, as make their pupils thorough in every fundamental branch of a medical education. Has it not been the case, that anatomy and surgery, theory and practice, and an item of obstetrics, to an undue extent have occupied attention to the almost entire neglect of chemistry, pharmacy, and other equally important branches? As veritable evidence of this course, have not many distinguished professors, in their departments, even within a few years, denied, almost entirely, the chemistry of the animal system? Never till the illustrious Liebig demonstrated the chemical laws of vegetable and animal nature, and placed them upon an immovable basis, did these champions, of bygone days, yield the point in controversy. Let none wonder, that empyrics are in the regular practice—that new schools after new schools of a different type, have arisen. Let none wonder that the animal system is not understood, and standard and important medicines are decried. Such will be the case, till, in the regular schools, a reform is not only attempted, but perfected. It is not equity to cast all the blame of the abuse of the practice, on innovators who arise of course, as long as the old and well tried systems of instruction are so truly defective. Whilst the light of chemical science is evident and increasing, is it not reasonable, that we be more attentive to its various parts, and give a fair proportion of *severe study* to its numerous principles, so intimately connected with an efficient practice of medicine. Do not infer, that because we sometimes teach chemistry, we appear especially as its advocate. For years, in and out of medical schools, we have been deeply impressed with these strange defects—we have even been witnesses of the channels in which medical instruction has run, and the singular neglects of the fundamentals alluded to. It is not our wish to depreciate anatomy and surgery, theory and practice, and obstetrics. These are exceedingly important in their place, but are limited, even if comparative anatomy be attached to the regular subject, while chemistry is altogether unlimited; nevertheless, we hesitate not to say, that such is the nature of the first mentioned subjects, that much more time ought to be appropriated to them, at least in private, than is usually assigned, but chemical science and its collaterals demand an equal share with its compeers.

To remedy the defect in question, it appears to us, more time ought to be appropriated, than is usually allotted to the attainment of the most difficult profession, in the catalogue of professions.

Of all the branches of a medical education, chemistry is, by fair comparison, the *most extensive* and the *most difficult* to be secured, and yet it receives by far the most superficial attention. Why this condition of things? Because so few are good theoretic, not to say practical chemists, and unless somewhat practical, in strict propriety, they cannot be esteemed chemists at all. To understand the science, necessarily implies practice, though we do not urge the latter to the extent of a teaching profession. It is from the small estimate put on chemistry, that faculties of respectable schools too frequently touch it lightly, on the supposition there is no time; than which nothing is more dangerous; inasmuch as it is preeminently a fundamental. If there really be not time, the difficulty might be remedied by requiring more of the pupil on entering a medical school. At least, a full and practical course of chemistry, as well as a more careful preparation on other subjects. Such a plan, might to some extent obviate the difficulty, or it may be, better obviated by a strict faculty, where every subject treated is most full and practical, by requiring of the learner, three full courses of lectures, and to pass a very rigid examination before graduation. If such were the fact, honor and success would usually attend the medical practice. Empyrics, as is meet, would pass into nonentity. The public would be relieved of medical blunders, and the herd of patent remedies, with the advertisements of *horrifing cures*, would no longer astound the ear, and newspapers might assume their legitimate office, as reporters of facts and principles. Rational curatives administered with knowledge, will be found adequate, and confidence secured to the medical practitioner. Even calomel, however defamers may abuse, if administered with a knowledge of the chemistry of the system, and the entire absence of alkalies, acids and table salt, by which most of the mischiefs result, is not only a safe, but the most efficient of medicines. We call upon dispensatories to reform some of their formulas in which calomel is an ingredient. If the proper knowledge be secured, and the practice careful, all will be safe. Thirty-eight years in the labratory, with many forms of experiment, to ascertain the action of calomel, have led to the above conclusion.

That our positions may be substantiated, it becomes us to look a little into the range of a science so fundamentally connected with the practice of medicine, whether in old or new schools. Whether they be Allopathic, Homœpathic, Neuropathic, Thermopathic, or any other athic or eclectic not enumerated, all are most seriously interested in the subject here noticed.

1. It is requisite, that the student of Dentistry, or of the regular practice, (we consider them one and the same,) or that others, specifically understand most of the chemical simples that enter into the numerous varied forms, which nature has so profusely distributed. The bubbling airs, from fermentation or putrifaction, the liquid lava, the limpid water, the mountain rock, the atom of dust, the spire of grass, the delicate vermis or animal, are all composed of *simple substances*, in a definite number of combining atoms, molecules or particles, so that a single particle more or less of each simple, will change the nature of the compound.

2. The power of affinity, regulated by many laws, demands most minute attention. On these laws and their operation, the success of all decompositions so indispensable in practice, depends. If in substances generally, the particles be held by the same power of attraction, there must be an end to all chemical research. The Great Architect, in infinite wisdom and goodness, has furnished to the corpuscles or atoms, of which all matter is constituted, different forces of attraction, that the power of affinity may be constantly illustrated in new forms or compounds of elementary matter. Millions of inorganic compounds arise naturally or artificially from the exercise of these laws. Many of the most productive manufactures, for their existence, depend wholly on the proper exercise of affinities, as sugar, salts, dyes, colors, calico printing, &c. Vegetable and animal nature, from the expansion of a leaf, or blow of a bulb, to the rounded limb and invigorated frame, is dependent on the same harmonious principle, for beauty and adaptation of parts.

3. The laws of Caloric demand the most earnest and delving investigation. In all their modifications, the bearing on the human subject makes them doubly interesting to the practitioner. Whether we view caloric in the hardest rock, in fluid water or elastic air, its condition is most curious. In its free or latent state, it proclaims the goodness of God. To know that the hardest ice contains, at the lowest calculation, one thousand degrees of this imponderable substance enchained; a portion of which, for the security of delicate vegetable and animal existence, is thrown out in every change of state, can not fail deeply to impress the mind with gratitude. Had it pleased the Almighty Governor to have constructed the varied objects of teeming nature, *without caloric*, the liquids and gasses, as we behold them, would have presented an appearance entirely different, and assumed a form approximating in hardness to adamant or to the solidity of the nether mill stone. It is caloric that gives us the limpid water, the invigorating air, the genial climate, the luscious fruits, the flush of health, in short, the most important blessings of organic and animated nature.

4. What shall be said of electricity, galvanism and magnetism, those mysterious imponderable substances, which flit through nature as with a magic wand. Singular and evanescent as they appear; what more stirring and powerful than their effects; what more important than their laws. We by no means affirm, as some late teachers have done, that electrical attractions and chemical affinities are identical, though they are certainly nearly related. The present state of the science does not authorize such a conclusion. Nor do we intimate that the galvanic fluid and nervous influence are the same, though experiments on digestion show something like a similar action. This may arise, and yet the nervous fluid be quite different from galvanism. Nor do we for one moment subscribe to the position gravely stated by recent innovators, that by the aid of galvanism "living animals" and "vegetables" without eggs or seeds, have been produced "from mere inorganic matter." (See Hollick's Neuropathy, page 135.) These conclusions are the reveries of inexperienced men.

Nor, finally on this subject, do we admit (which is taking the upper step in these delectable wanderings stated in so many words, or by fair implications in Hollick's Neuropathy, page 58,) the strong position deduced from certain galvanic experiment, "explaining to us," says the author, "the nature ot that *mysterious power*, which produces our *thoughts*, *feelings* and *actions*." In other words, galvanism constitutes the intelligent mind, and thus the thinking immortal soul is resolved into real matter; the Galvanic fluid: which exhibits without if or and, the essence of materialism. Strange as the fact may appear, it is nevertheless true, that a majority of modern innovators, as Phrenologists, Neuropathists, Mesmerists, &c., regularly push their systems into materialism. They mistake, or affect so to do, the true principles of philosophizing, and confound the properties of mind and matter, which are as essentially different as day and night, or as the Zenith and Nadir. We make the above statement, at the hazard of being charged with raising opposition *to progress* in science, by brazen-faced editors, who know little about real science, and less about evangelical truth.

After noticing what electricity, galvanism and magnetism are not; we feel free to consider them as modifications of one and the same influence, which is a subtile elastic fluid that pervades all matter, and is obedient to specific laws, many of which are understood and appropriated to the most useful purposes. It is this, that in a peculiarly modified form, produces the polarity of the needle, and exhibits in its operation many wonderful magnetic experiments. In another condition, it appears flashing beautifully through the sky, or in accumulated quantity descending in grandeur from the burdened cloud, and tearing in sunder the tall oak, or in accordance to the conducting substances, ascending in equal force from the earth to the negative cloud above—a fact often presented. In another form arising from acids and metals, or other substances on which acids have an action, or various substances acting chemically on each other, this influence is evolved and appropriated extensively in combustion, in decomposition, to the electrical telegraph, and is beginning to be somewhat used as an article of the materia medica, which fluid it becomes the practical physician and dental surgeon most carefully to investigate. In the general class of nervous diseases, its action is more efficient than any remedy in the catalogue, yet it is but little known and still less applied.

5. How can we, in these passing remarks, enumerate the thousand and one gasses which the different forms of matter in decomposition present. Some are simple with the exception of union with caloric, to which all gasses owe their elasticity and air-like form. Some are compounds, some are supporters of combustion, some are combustible, some are incombustible, some are supporters of life, some are poisonous, yet all are formed into one harmonious whole by the benificent Creator; not one could be spared without producing a breach in nature. What, from a partial view, we too frequently deem the greatest misfortune, really exhibits the greatest benevolence. The decomposition of vegetable and animal substances, chiefly into gaseous matter, though loathsome to the sight, and in some degree distressing, is fraught with infinite wisdom. From this source, the future races of both plants and animals are mainly sustained and perpetuated.* The gasses furnish the most brilliant experiments of the laboratory, and are of great value in various departments of medicine, in their direct or indirect influence on the animal subject. Oxygen, in its abundance, constitutes nearly one-half of the materials of the globe. Carbon, which enters so largely into animal and vegetable products, when in union with oxygen, forming carbonic acid, makes up one seventh of material things. Hydrogen, the lightest of gasses, which like its compeers, is strapped down in its condensed form, by an affinity that is truly incredible to finite creatures, is a very important element in the whole waters of the earth. If turned into its original state of gas, from all the waters of our great rivers, extended lakes, and wide oceans, the mighty mass, we conjecture, would fill a sphere equal in radius to the distance of Sirius, or twenty-one billions of miles, English numeration. How immense are the works of God!

6. If gas be thus imposing, how in all their bearings on the profession of the physician and dentist, shall we treat the long catalogue of *ide* compounds, including alkalies, alkaline earths, earths proper, metals, &c., of acids of varied form, simple and compound, sour and sweet. The distinctive character of sweet acids, consists in their power to neutralize *ides*, and form the class of substances known in chemistry, as *salts*—which ide, acid and salt compounds make up—the principal material of the materia

* It has been ascertained, that the chief support of vegetables, besides moisture, is carbonic acid and ammonia, which are furnished in a gaseous state by fermentation and putrifaction. The former is absorbed by the leaves of the tree or plant, and the latter by the roots, which subtract from the ammonia the requisite nitrogen. Animals depend on vegetables for support. The carnivora, that feed on animals, feed on such as subsist on plants, grasses or shrubs. Our position therefore is sustained, that fermentation and putrifaction, the great sources of carbonic acid and ammonia, sustain and perpetuate the future races of plants and animals.

medica. They furnish the standard drugs, the main reliance of every physician, either *Allopathic* or any other *athic*. Whether there be a crusade against some drugs, or all, or no crusade.*

*Much has been said in regard to poisoning by certain medicines. By some strange reasonings the public mind seems to be impressed, that all remedies are innocent, or exert no deleterious influence, except those derived from the mineral kingdom. Hence a peculiar prejudice exists, which is said to have shown itself in the legislature of a sister State. If so, it is time that the question be mooted: From which kingdom in nature, are the most virulently poisonous remedies derived? 1. In regard to the animal kingdom, little need be said, as no remedies of consequence are derived from this source. That poisons are to be found, in this connection, is true; as snake, scorpion, spider, wasp or bee poison. There are poisonous fish and flesh used as food, as poisonous sausages, or tainted, i. e. putrifying meat. Still none of these are employed as medicines. 2. In the vegetable kingdom, so abundant in remedies or medicines, we detect the most virulent, frightful and active poisons; vegetable preparations lay hold on the system at once, and so rapid is their operation, that counteracting influences can rarely be used. The preparation from peach leaves or kernels, from bitter almonds, from laurel or wild cherry, if concentrated, presents the most appalling results. Death, from this cause, usually occurs in a very few minutes. The same may be said of strychinne and brucine from nuxvomica, and a relative principle from the castor bean, of conine from hemlock, of hyoscyamine from hyoscyamus, of daturine from henbane, of attopine from atropa belladona, or even of the extract of belladona. Of the class of night shades, even of the sprouts of the common potato, solanine is a violent poison, of emetine from ipecacuana. We have seen reports of the very poisonous character of lobelia. Tobacco, so universally used as a luxury, is very poisonous. The extract from one pound of the well prepared article, is sufficient to destroy many lives, and in the shortest time. We could add considerably to the above catalogue, and the products mentioned are originally in the plants, seeds, or trees, and are not made, but only separated and collected, by chemical process. The poisonous character of alcohol results from fermentation, and not from the operation of the still. Hence, fermented liquors, containing an actual portion of alcohol, as beers, ciders, wines, &c., are inconsistent with teetotalism. 3. From the mineral kingdom are derived many substances, which, when used in excess, or without full knowledge of their chemical action, produce poisoning. The oxides and salts of lead, copper, zinc, arsenic, mercury, &c., and chlorides are in their proper doses, good remedies—but when administered without regard to chemical relations, are sometimes highly injurious. The poisoning, however, is slow, in comparison with the energetic action of vegetable principles. In balancing between vegetable and mineral medicines, if we be allowed to give an opinion, the danger of injurious effects to the human constitution, is ten-fold greater from vegetable than mineral medicines.

We would make one remark concerning the preparation of Homæpathic medicines, so much insisted on by the advocates of that type of administration. It is contended, that the efficiency of their small and divided doses, results from their medicines being prepared in a particular way. Now, it is known, that those practitioners are not over notorious for chemical knowledge, and that they profess to use many allopathic drugs in divided doses. We wish to know, if their calomel or quinine (we give these

From what has been stated, we apprehend by the student of dentistry or medicine, some fair conclusions may be drawn of the extent and importance of our subject. We are, however, scarcely over the threshhold. The most important points of our discussion remain untouched and time fails. Only a few passing words can be employed on our seventh division, comprising vegetable and animal chemistry.

A few years since, this subject was almost untouched, and when approached, it was in such a way, as to furnish little light. Very lately, such men as Liebig, Dumas, Mulder, Sherer, Jones, Will, Gregory, Boussingault and others, so effectually surmounted the difficulty and cleared the way, that a harvest of principles has been collected, or a mine of gems has been uncovered, and to some extent appropriated.

The lamp of Chemistry, has so illumined the mysterious path of organized nature, as not only to ascertain the proper pabulum, but the functions of many hidden parts of the vegetable and animal systems. 1. Vegetable kingdom—From the seed to the perfect fruit, the office of the roots, the stalk, the limbs, the bark, the leaves, the flower, &c., has been minutely, anatomically, and chemically examined. The food of plants, and the manner in which that food is secured and assimilated, under the superintendance of the principle of life, by the immortal Liebig, have been duly detected and illustrated. It is ascertained that the vegetable takes up no nourishment, except that which is dissolved in water, or absorbed in a gaseous state by the vegetable parts. That, after the plantule, sustained by a chemical provision, has arisen a small distance from the ground, its roots draw up salts, ammonia and carbonic acid, but its chief support is derived through the medium of the leaves, from carbonic acid, always present in fertile ground in quantities for a supply. That humus, geine, or vegetable mole, on which soil depends for its fertility, only acts by conducting the only as examples) can be, in preparation, any other than literally *chemical calomel*, or *chemical quinine*. To us, the asservation of peculiar preparation, has always appeared a humbug. Even the sugar of milk has considerable chemical action on many medicines.

process of decay or eremacausis and evolving carbonic acid, which the roots eagerly absorb, together with the requisite salts of the manure—that ammonia is one of the most important items in the soil, derived chiefly from animal putrifaction—that this substance is decomposed, and its nitrogen essential to the tissues, flowers and fruits, is appropriated, while the hydrogen of the ammonia in the living organism is combined with oxygen in the form of water. By the power of the leaves, immense amounts of carbon, from carbonic acid, are assimilated and appropriated, so as to constitute the solid or principal part of the material of each elaborated plant or tree, as well as of the seed or fruit. Here we see proceeding a most beautiful and harmonious order of nature. Fermentation, the breathing of animals, volcanic fires and combustion in all forms, these combined, absorb immense quantities of oxygen from the air, and yield carbonic acid. From this air, vitiated as above, the carbonic acid is rapidly taken up, by the leaves of vegetation, its carbon is separated by the chemical agency of light, and assimilated by the power of the vegetable. In this great process of nature, we witness a great amount of carbonic acid taken in, and a part appropriated, and a continued flow of pure oxygen thrown out, so as to restore to a healthful condition the air which we breathe. In this manner the beneficent Creator has made vegetable and animal natures contribute to mutual support and health, in more ways than one. In regard to the nutricious parts of plants, they are so arranged as exactly to suit the wants of the animal.

The vegetable, as the lowest in the scale of organization, draws its food from inorganic nature, while the animal derives his from organized vegetables, showing in this particular, a character strikingly superior to the latter, and this is realized not only in animals which live on vegetables, but also in such as prey on their kind, inasmuch as the animals eaten derive their support from grasses, seeds and fruits. In either case from organized substances.

2. Animal kingdom: again if we turn to animal chemistry, every fact is important. We first notice an embryo part, then an infant or elementary animal. Peculiar organs of nutrition, assimilation, increase and production. For the infant, in many orders of animals, (where it is wanting, the supply is from expedient of nature,) we find a pabulum of very strange constructions in the milk of the parent. For the bones a salt ready prepared. For the tissues, constituting muscle, membrane, tendon, nerve, &c., substances precisely suited to their condition. When the animal attains maturity, food in various forms is found, rich in carbon and an ample portion of nitrogen, required for the compacting of animal flesh. The mouth masticates the food, and the stomach receives it—there a regular chemical process is conducted. The food becomes chyme in the first play of affinities, by a second action, in which the gall and pancreatic secretions perform their part—the chyme is changed to chyle—this is absorbed by the lacteals, and carried to the circulation, when with the venous blood the former is sent to the lungs. This fluid absorbs its amount of oxygen, and gives out carbonic acid. As arterial blood, it is sent to every part of the system, to supply by secretion and assimilation, the waste of the animal. Thus is vital action sustained, and locomotion regulated, by the will of the creature. The wonderful fact is here developed, that the elements of blood are fibrine albumen and salts, the very materials for the support of animal flesh and bone in all forms, the milk furnishing another principle, called caseine, to complete the full supply of increase and repair in the system. We detect in the vegetable, whether grass, seeds or fruits, precisely the same elements as above; required for the support of the blood of the animal, viz: vegetable fibrine, vegetable albumen, and vegetable caseine, containing nearly the same elements as in the blood of animals. Hence vegetable nature has only to be elaborated, to extract, by appropriate apparatus, the elements of blood, and the whole supply for the animal is secured. Who does not see, in the adaptation of vegetable and animal principles to each other, by a ready conversion of one unto the other, the wonderful goodness and benevolence of Deity? If we trace the elements of blood still further, we are carried up to the *one substance*, called by chemists proteine, similar in composition to fibrine, albumen and caseine. The combining atoms of simples differ very little in these elements of blood—this one substance is prepared and adapted to form skin, hair, muscle, nerve, bone, sinew, &c. Here we discover a simplicity which every where characterizes God's creation.

Again, if we examine the blood in its motion, we find an oxide of iron, which in the veins, is a prot or first oxide, but in the arteries, after the action of the lungs, by absorbing oxygen, is changed to a per or higher oxide of iron, and constantly alternating in each situation, to suit the required vital action.

Here, too, the same glorious simplicity is manifest, as already shown. From these particulars may we not infer the food, situation, and other circumstances best adapted to health, and the increase and diminution of the animal, the causes of disease, the decay of parts, the remedies appropriate, and thus furnish to the dentist the precise nature of flesh and bone, the causes of Eremacausis, or decay in the teeth, the best substances for filling, wherever caries has made its unpleasant ravages? The forms of matter for artificial teeth, are presented, and even teeth and gums, where accident or disease has occasioned their absence. These articles, by the hand of the practical chemist, are now successfully made.

As far as our time serves, and God permits, we shall not fail to hold up to view the great principles hastily sketched, even to the end of our course.

From what has been imperfectly and concisely stated, we think it is abundantly evident, that the *prominent and principal defect* in a medical and dental education, consists, chiefly, in the neglect of the great and difficult department of Chemistry. While other subjects ought to be well studied; let this peculiarly imposing part be properly brought up; and empyricism and quackery must soon retire, and a safe and successful use of the most defamed medicines will soon be realized, and the profession will be made doubly useful and honorable.

These hasty, and necessarily superficial remarks, are seriously addressed to the students of Dental Surgery, that they may examine for themselves, and not improperly draw the conclusion, as we have known too many to do, that Chemistry is of small importance in the profession of Dental Surgery.

